# Bail-Out Techniques in Percutaneous Intervention for Ellis Grade III Coronary Perforation in Left Main Distal Bifurcation Lesions

**DOI:** 10.1016/j.jscai.2023.100609

**Published:** 2023-03-11

**Authors:** Kensuke Takagi, Ruka Yoshida, Tomoyuki Fujita, Teruo Noguchi

**Affiliations:** a Department of Cardiovascular Medicine, National Cerebral and Cardiovascular Center, Osaka, Japan; b Department of Cardiology, Japanese Red Cross Society Nagoya Daini Hospital, Nagoya, Japan; c Department of Cardiac Surgery, National Cerebral and Cardiovascular Center, Osaka, Japan; d Department of Advanced Cardiovascular Medicine, Graduate School of Medical Sciences, Kumamoto University, Kumamoto, Japan

**Keywords:** Bail-out, coronary perforation, covered stent, Ellis grade III, left main coronary artery

## Abstract

The left main (LM) coronary artery stenosis is associated with high morbidity and mortality and has traditionally been treated with coronary artery bypass grafting. However, in recent years, advancements in device technology and adjunctive pharmacotherapy have led to the widespread use of percutaneous coronary intervention (PCI) as a treatment for unprotected LM lesions. Despite this, LM lesions are often complex, involving distal bifurcation and heavy calcification, which increases the risk of coronary perforation (CP) during PCI. In addition, the use of rotational or orbital atherectomy in severely calcified LM bifurcation lesions carries a higher risk of complications and in-hospital mortality than that in non-LM lesions. CP is a rare but potentially fatal complication of PCI, particularly in cases of Ellis grade III (CP-G3), with a high rate of cardiac tamponade and mortality. The management of CP-G3 in LM distal bifurcation lesions is challenging and requires specialized techniques. This article presents a flowchart of bail-out strategies for CP-G3 in LM distal bifurcation lesions and provides detailed procedures for each technique. Furthermore, we highlight the challenges and limitations of each technique, requiring careful management when CP-G3 occurs in LM distal bifurcation lesions.

## Highlights

• Management of Ellis grade III perforation in left main bifurcation is challenging.
• Hemostasis and flow maintenance in 2 epicardial coronary arteries are necessary.
• Surgical repair of left main perforation is complex because of poor viewpoint.
• Complete hemostasis by covered-stent at any cost, followed by bypass surgery.

## Central Illustration

**Figure:**
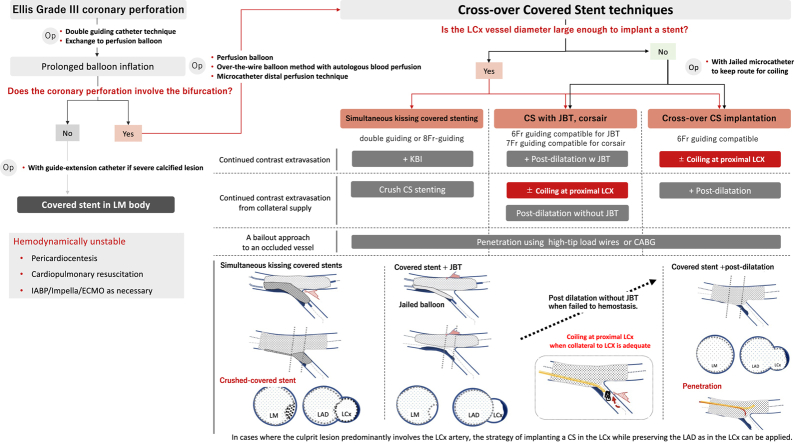

## Introduction

The left main (LM) coronary artery stenosis is associated with high morbidity and mortality and has traditionally been treated with coronary artery bypass grafting.^1^ However, device technologies and adjunctive pharmacotherapy advancements have led to the widespread indication of percutaneous coronary intervention (PCI) as a treatment for unprotected LM lesions over the past decade.^2^, ^3^, ^4^ Nevertheless, LM lesions are rarely focal, commonly involve distal bifurcation, and are frequently heavily calcified.^5^, ^6^, ^7^ Therefore, coronary perforation (CP) potentially occurs in severely calcified LM lesions during PCI. Furthermore, in severely calcified LM bifurcation lesions requiring lesion modification, the rate of PCI using rotational or orbital atherectomy ranges from 6.0% to 10.6%.^8^, ^9^, ^10^ However, these devices for LM lesions are reported to carry a higher risk of complications and in-hospital mortality than for non-LM lesions.^11^, ^12^, ^13^, ^14^, ^15^

In general, CP is a rare but potentially fatal complication of PCI, especially in Ellis grade III (CP-G3) cases, which also have a high rate of cardiac tamponade and mortality.^12^^,^^16^^,^^17^ When CP-G3 occurs in LM bifurcation lesions, its management is challenging, and its hemodynamics are more prone to collapse. Although several studies have reported the management of CP,^11^^,^^13^^,^^16^^,^^18^, ^19^, ^20^, ^21^, ^22^, ^23^, ^24^, ^25^, ^26^, ^27^, ^28^ to the best of our knowledge, there have been no reports focusing on the management of CP-G3 involving LM distal bifurcation lesions. In this study, we designed a flow chart of the bail-out strategies in the setting of CP-G3 to provide a comprehensive bail-out strategy for specialized LM distal bifurcation lesions. Furthermore, detailed procedures for the cases corresponding to each technique are summarized, and the limitation of each technique is clarified.

## The incidence and management of CP

The details of CP are summarized in Supplemental Table S1. The overall prevalence of CP ranges from 0.2% to 0.9%.^11^^,^^13^^,^^18^, ^19^, ^20^, ^21^, ^22^, ^23^, ^24^, ^25^, ^26^, ^27^, ^28^ The average age of patients was between 66.5 and 75.4 years, with a male predominance of 57.9% to 74.9%. The prevalence of acute coronary syndrome varied from 4.8% to 75.0%. In comparison, the frequency of heparin reversal ranged from 1.3% to 45.1%, which indicated that the data were heterogeneous and different based on the country and enrolled year. Recently, the Glasgow Natural History Study of Covered Stent Coronary Interventions Study demonstrated that the CP was increasing over the years and perforation severity was linearly associated with procedural mortality (a median of 2.9-year follow-up): Ellis I (0%), Ellis II (1.7%), Ellis III/IIIB (21%) (*P* < .001).^18^ In general, Ellis grades I and II perforations are usually managed conservatively without specific treatment. However, the management should be more careful when CP is in the LM lesions. Notably, the systematic review also shows that this incidence of CP-G3 increased over time and accounted for 43% of all CP.^12^ Although CP-G3 is rare, ranging from 0.1% to 0.4%, tamponade progresses rapidly and is associated with the requirement for urgent coronary artery bypass grafting and a high mortality rate^12^^,^^13^^,^^19^^,^^20^^,^^23^^,^^25^^,^^27^^,^^28^ (Supplemental Table S1).

A common initial approach for hemostasis in CP is prolonged balloon inflation. The position of the inflated balloon is changed slightly with each balloon dilation; contrast injection simultaneously with balloon dilation can help identify the perforation site. In cases of hemodynamic instability, urgent pericardiocentesis, intra-aortic balloon pumping, Impella devices, and extracorporeal membrane oxygenation should be considered. Anticoagulation should not be immediately reversed with the wire and balloon in the vessel during attempted perforation hemostasis. Although partial reversal is controversial, an immediate reversal could lead to thrombosis throughout the entire vessel leading to a higher degree of mortality than the perforation itself. The use of a perfusion balloon catheter might be an effective option because the balloon can remain inflated for an extended period without impeding the coronary flow at the distal segment. When a perfusion balloon is not available, the over-the-wire balloon method with autologous blood perfusion^29^ or the microcatheter distal perfusion technique^30^ are alternative strategies. However, the exact recommended ballooning time to achieve hemostasis needs to be clarified because the mechanism of CP also affects hemostasis. When the CP cannot be weakened after several balloon attempts, this is the time to change the strategy of using a covered stent (CS). CS deployment with a polytetrafluoroethylene-CS and the possibility of emergent surgical repair may be considered when balloon inflation fails to achieve hemostasis.

A CS is the final and effective option for percutaneous bail-out for CP; the success rate of hemostasis after CS implantation ranges from 73.3% to 93.8%.^19^^,^^20^^,^^22^, ^23^, ^24^^,^^27^^,^^28^^,^^31^, ^32^, ^33^, ^34^ CS effectiveness has been limited by poor deliverability with delivery success rates of ∼80%.^23^^,^^34^ Recently, new CSs with improved deliverability have launched. They include a new-generation single-layer polytetrafluoroethylene-CS and an electrospun polyurethane-CS.^32^^,^^33^^,^^35^ Therefore, although the management of CP after PCI for LM bifurcation lesions is still challenging, hemostasis using CS for these lesions is worth attempting and is more accessible to interventionists.

The treatment of CP involving LM bifurcation lesions entails 2 challenging issues: hemostasis and maintenance of coronary flow in both epicardial coronary arteries are necessary. First, the LM bifurcation lesion at a high risk of CP is usually composed of severely calcified lesions that require lesion modification; thus, CS delivery is usually difficult, and complete hemostasis is not easy because of the underexpansion of the CS. Second, LM bifurcation lesions have a large side branch (SB); furthermore, the location of the CP makes complete hemostasis more difficult. Loss of the SB flow occurs in 2.9%–12.8% of patients.^11^^,^^20^^,^^26^^,^^33^ This SB sacrifice increases in cases of CP requiring CS implantation or CP involving LM bifurcations. Although surgical repair is necessary for patients with failed percutaneous hemostasis, mortality after surgical repair is high, with in-hospital mortality rates of 14.3%–75%.^13^^,^^19^^,^^23^^,^^27^^,^^28^^,^^34^ A surgical repair of the LM artery perforation sites is complex because of the poor viewpoint behind the pulmonary artery, complicated maneuvers, and limited time, especially when prompt repair is required for active bleeding. Therefore, a realistic scenario would be to achieve complete hemostasis by percutaneous CS implantation at any cost, followed by bypass surgery or a percutaneous approach to the occluded coronary artery, if hemodynamic instability remains after complete hemostasis.

## Summarized patient characteristics with CP involving LM bifurcation

There is a limited number of studies focused on CP involving LM lesions. The sole report on this subject was from the British Cardiovascular Intervention Society registry.^11^ The British Cardiovascular Intervention Society registry reported 96 patients with CP involving LM lesions from 2007 to 2014, with an incidence rate of 0.9% and a significant increase in unprotected LM-PCI every year, resulting in an increasing trend in the incidence of CP involving LM bifurcation. A surgical repair was not an option, and the incidences of SB sacrifice and in-hospital mortality were 12.8% and 19.3%, respectively. In addition, a representative bail-out case regarding CP of the LM artery is summarized in Table 1^36^, ^37^, ^38^, ^39^, ^40^, ^41^ from a literature search of MEDLINE. The search was performed in English using the keywords: coronary perforation, left main, and/or percutaneous coronary intervention, including their subheadings and synonyms. The search was performed in October 2022, yielding 169 results. After titles and abstracts were reviewed, 6 case reports describing detailed procedures were found, including 7 patients with CP involving distal LM bifurcation lesions.

**Table 1:** Characteristics of 7 patients with coronary perforation involving left main lesions according to representative techniques.

No.	Age	Sex	CP location	Cause of CP	Ellis grade	No. of guiding catheters	MCS	Bail-out strategy	Details of bail-out	Pericardiocentesis	Heparin reversed	Surgical repair	In-hospital mortality	Follow-up	Reference
1	83	Female	LM-bifurcation (lateral side)	Rota burr 1.5 mm	III	Single	IABP	CS in crossover LM-LAD jailed balloon	GraftMaster 3.0 × 19 mm with jailed balloon 1.5 × 15 mm	No	No	No	No	6 mo	Takagi et al^40^
2	79	Female	LM-bifurcation (Carina)	DES (LM-LAD) with 4.0/20	III	Double	None	Simultaneous kissing covered *stents*	Perfusion, SKCS GraftMaster	No	No	No	No	6 mo	Moriyama et al^41^
3	91	Male	LM-bifurcation (Carina)	DES (LM-LCx) with 4.0/28	III	Single	Impella CP	CS in crossover LM-LCx; LAD penetration	PK Papyrus 3.5 × 16 mm (LM-LCx) LAD penetration with CP12	No	NA	No	No	6 mo	Adusumalli et al^38^
4	75	Female	LM-ostium/body	POBA 1.25 mm	III	Single	None	CS in crossover LM-LAD; LCx penetration	PK Papyrus 3.5 × 20 mm (LM-LAD), LCx penetration with CP12	No	NA	No	No	6 mo	Werner and Ahmed^37^
5	88	Male	LM-bifurcation (LAD side)	DES (LM-LAD) with 3.5/18	III	Single	IABP/ECMO	CS in crossover LM-LAD; LCx penetration 4 d after PCI	GraftMaster 3.5 × 16 mm (LM-LAD), LCx penetration with CP8-20	Yes	NA	No	No	NA	Taniguchi et al^36^
6	84	Female	LM-bifurcation (LCx side)	Rota burr 1.75 mm	III	Single	IABP	CS in crossover LM-LCx	GraftMaster 4.0 × 16 mm	Yes	NA	No	No	6 mo	Gamma and Thomas^39^
7	78	Female	LM-bifurcation (LCx side)	Rota burr 2.0 mm	III	Single	IABP	CS in crossover LM-LCx	GraftMaster 4.0 × 16 mm	No	NA	No	No	2 y	Gamma and Thomas^39^

CP, coronary perforation; CS, covered stent; DES, drug-eluting stent; ECMO, extracorporeal membrane oxygenation; IABP, intra-aortic balloon pumping; LAD, left anterior descending artery; LCx, left circumflex coronary artery; LM, left main trunk; MCS, mechanical circulatory support; SKCS, simultaneous kissing covered stents.

In these 7 patients with CP involving LM distal bifurcation lesions, the leading cause of CP was postdilation, stent implantation, and rotational atherectomy. Most patients required intra-aortic balloon pumping or Impella (Abiomed) because of hemodynamic instability. One was treated with CS with the jailed balloon technique (JBT), 1 with simultaneous kissing covered stent (SKCS), and 5 with crossover-CS implantations. Of these 5 patients with crossover-CS implantation, penetration of CS using CONFIANZA Pro (ASAHI INTECC) wire was conducted in 3 patients and succeeded in recanalizing the jailed coronary artery, whereas the remaining 2 patients were treated conservatively because of their stable hemodynamics.

## Selecting the approach for CP using our flow chart

To appropriately select the intervention for CP, we designed a flow chart (Central Illustration). In this figure, we present different case scenarios and their respective managing recommendations. When angiography or imaging reveals a CP site that does not involve a bifurcation, CS implantation is a favorable option after prolonged balloon inflation fails to achieve hemostasis at the CP. When the CP site is close to the bifurcation, protection of the SB by thoroughly inflating the balloon could guide the indwelling-CS to an optimal position in the distal main vessel. When the CP site is not identified in bifurcation lesions, a balloon must be repeatedly inflated and moved from the distal main vessel of the bifurcation lesion; a contrast medium needs to be simultaneously injected to identify the site of the perforation.

**Central Illustration:**
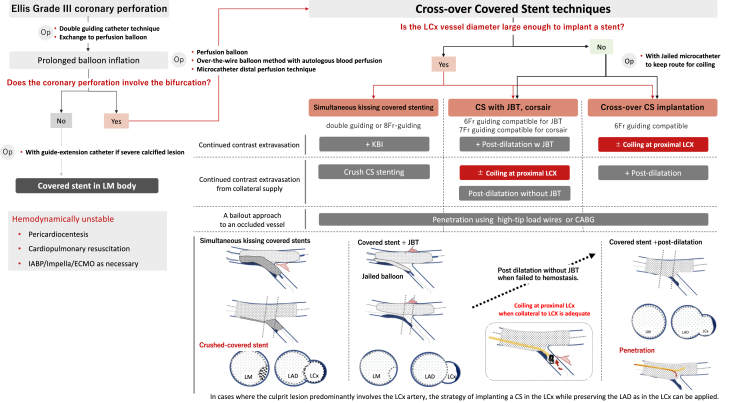
**Flow chart of bail-out strategy for coronary perforation.** The flow chart is based on a case series to understand the comprehensive bail-out strategies for specialized LM bifurcation lesions. Covered stent with jailed balloon technique and simultaneous kissing covered stent are recommended before the crossover-covered stent implantation over the LM bifurcation to increase the probability of saving coronary flow. ECMO, extracorporeal membrane oxygenation; IABP, intra-aortic balloon pumping; JBT, jailed balloon technique; KBI, kissing balloon inflation; LAD, left anterior descending artery; LCx, left circumflex coronary artery; LM, left main coronary artery.

Crossover-CS implantation is the most widely used technique for completely covering the CP site involving the LM distal bifurcation.^36^, ^37^, ^38^, ^39^ However, there are 2 alternative options that can preserve left circumflex coronary artery (LCx) supply even after crossover-CS implantation for the left anterior descending (LAD) artery. One such option is using a jailed balloon or a microcatheter, which is adequate to maintain the LCx flow effectively.^40^ This technique is considered an option when CP is present on the contralateral side of the bifurcation. Typically, postdilation in conjunction with JBT is sufficient to achieve hemostasis. However, when the CS fails to achieve hemostasis, further postdilation of the CS may be necessary. Before the LCx route is fully compressed, it is imperative to evaluate the risk of residual bleeding, such as a collateral blood flow. In case of residual bleeding from the collateral flow, microcoil embolization at the LCx ostium is added and required. Notably, this technique can achieve complete hemostasis by preventing endoleaks from collateral vessels, even after CS implantation. Conversely, in cases where the culprit lesion predominantly involves the LCx artery, this strategy can be applied for CS implantation for the LCx, whereas preserving the LAD as previously described for the LCx.

Second, the double-perfusion balloon technique through a double-guiding catheter effectively manages the CP in bifurcation lesions involving a large LCx. In particular, a double-guiding approach is a viable technique when the severity of CP leads to excessive bleeding. Despite only limited retrospective data, this technique might decrease the risk of periprocedural mortality due to the prevention of coronary bleeding during exchanging of catheters and devices.^42^ Furthermore, given that the SKCS technique requires a double-guided or 8-F catheter, the double-guided approach is a reasonable bridge to step up to the SKCS technique. After kissing balloon inflation (KBI), the SKCS technique using 2 CS is an effective option using the double-guiding technique.^43^ When the SKCS technique fails to achieve hemostasis after several attempts of KBI, and a leak is detected, the CS related to persistent bleeding could be compressed by postdilation through the other CS, resulting in complete hemostasis in exchange for an SB sacrifice.

When the occluded coronary artery interferes with coronary circulation after the crossover CS or SKCS, penetration using high-tip load wires toward the occluded artery as a marker for the jailed wire might be a good option regardless of the CS technique.^36^, ^37^, ^38^ As a last resort, a coronary bypass graft for the occluded artery is an option when the occluded artery covers a large territory of myocardium or hemodynamics are unstable due to acute heart failure because of large posterior wall ischemia.

## Discussion

This report identified several important points from the literature. First, because the indications for PCI for complex lesions have become more widespread and the necessity of lesion modification has increased, the incidence of CP-G3 has also increased.^12^^,^^18^ Second, although CS implantation is the ultimate option, the success rate of hemostasis after CS implantation is limited (73.3%–93.8%).^19^^,^^20^^,^^22^, ^23^, ^24^^,^^27^^,^^28^^,^^31^, ^32^, ^33^, ^34^ Furthermore, the difficulty of CS implantation is accentuated in the setting of LM bifurcation owing to the need to sacrifice a certain percentage of coronary flow in the LCx or LAD, whose territory is large enough to lead to hemodynamic compromise.^11^^,^^20^^,^^26^^,^^33^ Third, the overall postoperative mortality rate of CP-GP3 is 14.3%–75%.^13^^,^^19^^,^^23^^,^^27^^,^^28^^,^^34^ CP-GP3 localized to the LM bifurcation may be more challenging owing to poor visibility behind the pulmonary artery, complicated operation, and limited time, especially when severe bleeding and rapid repair are required. Finally, to the best of our knowledge, we present the first flow chart of a bail-out strategy for CP involving LM distal bifurcation, focusing on the complete hemostasis of the bail-out Ellis grade III CP. In this flow chart, consideration of alternative options, such as CS with JBT and SKCS, are recommended before conducting the crossover-CS implantation over LM bifurcation lesions because these techniques may increase the probability of saving the coronary flow of the LCx and that of the LAD. A comprehensive understanding of a dedicated bail-out strategy for specified LM distal bifurcation leads to better management of CP-G3, even involving LM distal bifurcation lesions.

## Limitations

This study has several limitations. First, the limited number of patients with CP-G3, including those with LM lesions, does not allow us to assess the adaptability of this flow chart in a real-world clinical practice. Second, anticoagulant reversal during CP bail-out is controversial.^44^ Anticoagulant reversal strategies are not discussed because of limited data. Although a heparin reversal for CP caused by wire in distal lesions and after the achievement of complete hemostasis is effective, heparin reversal for active CP in proximal lesions should be carefully considered, considering that intraprocedural stent thrombosis can be fatal. Finally, this study did not adequately assess differences between a traditional CS and a new-generation CS. Further dedicated studies in this specific field are needed to solve these issues.

## Conclusions

Because LM distal bifurcation lesions are frequently heavily calcified, and contemporary PCI requires lesion modification for these complex LM lesions, Ellis grade III CP potentially occurs in these lesions. Therefore, it is essential to have a comprehensive bail-out strategy for this fatal complication during complex LM-PCI. Our bail-out flow chart can help cardiovascular interventionists to decide which bail-out technique is the most appropriate for an Ellis grade III CP.
