# Supplementary material for: Bail-Out Techniques in Percutaneous Intervention for Ellis Grade III Coronary Perforation in Left Main Distal Bifurcation Lesions
Source: J Soc Cardiovasc Angiogr Interv. 2023 Mar 11;2(3):100609. doi: 10.1016/j.jscai.2023.100609 (PMC11307895; doi:10.1016/j.jscai.2023.100609)
Supplement: Supplemental Table S1 [file mmc1.docx]

**Supplemental Table 1. Characteristics of patients with coronary artery perforation according to each report.**

|  | **Characteristic** | **Lesion type** | **CP vessel** | **Cause of perforation** | **MCS/Heparin** | **Treatment strategy** | **Success rate** | **Outcome** | **Report, year,** |
| --- | --- | --- | --- | --- | --- | --- | --- | --- | --- |
| **Registry: GNOCCI Study** | Age: 69.0 | CTO:19.3% | LM: 3.5% | Wire: 12.3% | Pericardiocentesis acute: 40.4% | Prolonged Balloon inflation: 86.0% | Overall success: n. a | Final TIMI grade 0-1: | J Am Heart Assoc. 2022;11:e024492. |
| Enrolled period: 2001-2019 | Male: 57.9% | Bifurcation: n. a | LAD: 56.1% | POBA: 8.8% post:36.8% | Pericardiocentesis delayed: 3.5% | Stent implantation: n. a | After prolonged BI: n.a: | Cardiac shock: 49.1% |  |
| CP: 0.37% (161/43343), Ellis G3: 0.13% | DM: 21.1% | Calcification: 66.7% | LCx: 10.5% | Stent: 28.1% | IABP: 7% | Covered stent: 63.2% | After CS implantation: n. a | 24 hours mortality: 21.1% |  |
| Enrolled number: G3 57 patients | ACS: 57.9% | Type B2C: n. a | RCA: 17.5% | Rotational: 12.3% | ECMO: n. a | Coil embolization:1.8%, Fat: 1.8% | Surgical repair success: n. a | Acute stent thrombosis: n. a |  |
| Enrolled number: G1/2 104 patients |  |  | Others:10.4% |  | Heparin reversed: 17.5% | Surgical repair: 14.0% |  | Periprocedural MI: 22.8% |  |
| **Registry: G3-CAP (Cardio group VI)** | Age: 70.5 | CTO:22.3% | LM: 3.1% | Wire: 0% | Pericardiocentesis: 52.1% | Prolonged Balloon inflation: 73.2% | Overall success: 90.7% | Final TIMI grade 0-1: 27.3% | Am J Cardiol. 2021 Mar 15;143:37-45. |
| Enrolled period: 1998-2019 | Male: 74.9% | Bifurcation: n. a | LAD: 49.5% | POBA: 66.0% | IABP: 12.4% | Stent implantation: 14.4% | After prolonged BI: 2.8% | Cardiopulmonary resuscitation: 13.9% |  |
| CP: n. a | DM: 33.2% | Calcified lesion: 43.3% | LCx: 19.1% | Stent: 22.2% | ECMO: n. a | Covered stent: 64.4% | After CS implantation: 87.2% | In-hospital mortality: 7.2% |  |
| Device related Ellis G III: 0.18% (194/106592) | ACS: 43.7% |  | RCA: 24.7% | Rotational: 5.2% |  | Coil embolization: 5.2% | Surgical repair success: 46.1% | Acute stent thrombosis: 3.1% |  |
| Enrolled number: 194 patients |  |  | Others: 3.6% | Directional: 1.0% | Heparin reversed: 37.6% | Surgical repair: 6.7% |  | Periprocedural MI: 28.3% |  |
| **Registry: BCIS** | Age: 73.8 | CTO: 17.2% | LM: 100% | Wire: n. a | Pericardiocentesis: 25.0% | Prolonged Balloon inflation: n. a | Overall success: n. a | Slow flow: 3.2% | Catheter Cardiovasc Interv. |
| Enrolled period: 2007-2014 | Male: 60.4% | Bifurcation: n. a | LAD: 0% | POBA: n. a | IABP: n. a | Stent implantation: n. a | After prolonged BI: n. a | In-hospital mortality: 19.3% | 2021 Feb 1;97(2):E179-E185. |
| CP: 0.9% (96/10373 in LM-PCI) | DM: 25.6% | Calcification: n. a | LCx: 0% | Stent: n. a | ECMO: n. a | Covered stent: n. a | After CS implantation: n. a | Shock: 13.5% |  |
| Ellis G3: n. a | ACS: n. a | Type B2C: n. a | RCA: 0% | Rotational: 30.7% |  | Coil embolization: n. a | Surgical repair success: n. a | Periprocedural MI: 10.2% |  |
| Enrolled number: 96 patients |  |  | Others: 0% | Laser: 1.3% | Heparin reversed: n. a | Surgical repair: 0% |  | SB loss: 12.8% |  |
| **Registry: CIRC-8 study** | Age: n. a | CTO: 36.6% | LM: 6.1% | Wire: 67.0% | Pericardiocentesis: 13.4% | Prolonged Balloon inflation: 47.6% | Overall success: 89.0% | Final TIMI grade 0-1: | Cardiovasc Interv Ther. 2020 Oct 9. |
| Enrolled period: 2003-2014 | Male: 67.0% | Bifurcation: n. a | LAD: 35.4% | POBA: 23.1% | IABP: 17.1% | Stent implantation: 2.4% | After prolonged BI: n. a | Cardiopulmonary resuscitation: n. a |  |
| CP: 0.26% (82/31262) | DM: 40.2% | Calcification: n. a | LCx: 8.5% | Cutting balloon: n. a | ECMO: 7.3% | Covered stent: 18.3% | After CS implantation: 73.3% | In-hospital mortality: 12.2% |  |
| Ellis G3: 0.09% (29/31262) | ACS: 35.4% | Type B2C: 96.3% | RCA: 50.0% | Stent: 6.1% |  | Coil embolization: 2.2% | Surgical repair success: 25% | Acute stent thrombosis: n. a |  |
| Enrolled number: 82 patients |  |  | Others: n. a | Rotational: 3.7% | Heparin reversed: 45.1% | Surgical repair/CABG: 11.0% |  | Periprocedural MI: n. a |  |
| **Registry: BeGraft** | Age: 71.1 | CTO: 29.5% | LM: 0% | Wire: 16.4% | Pericardiocentesis: 26.2% | Prolonged Balloon inflation: n.a | Overall success: 96.7% | Final TIMI grade 0-1: n. a | Catheter Cardiovasc Interv. |
| Enrolled period: 2013-2017 | Male: 73.8% | Bifurcation: 34.4% | LAD: 54.1% | POBA: 59.4% | IABP: n. a | Stent implantation: n. a | After prolonged BI: n. a | Cardiopulmonary resuscitation: n. a | 2019 Apr 1;93(5):912-920. |
| CP: n. a | DM: 19.6% | Calcification: 23.0% | LCx: 16.4% | Cutting balloon: n.a | ECMO: n. a | Covered stent: 100% | After CS implantation: n. a | In-hospital mortality: 8.2% |  |
| Ellis G3: 83.6% of 61 patients | ACS: 23.0% | Type B2C: 82.0% | RCA: 23.0% | Stent: 25.0% |  | Coil embolization: 0% | Surgical repair success: n. a | Acute stent thrombosis: 0% |  |
| Enrolled number: 61 patients using new CS |  |  | Others: 6.6% | Rotational: 0% | Heparin reversed: n. a | Surgical repair: 4.9% |  | Periprocedural MI: 31.1% |  |
| **Registry: NCDR** | Age: 71.0 | CTO: 21.0% | LM: n. a | Wire: 42.6% | Pericardiocentesis acute: 21.6% | Prolonged Balloon inflation: 70.6% | Overall success: n. a | Final TIMI grade 0-1: 20.5% | Catheter Cardiovasc Interv. |
| Enrolled period: 2009-2016 | Male: 63.0% | Bifurcation: 17.0% | LAD: 20.6% | POBA: 29.4% | Pericardiocentesis overall: 29.4% | Stent implantation: 13.7% | After prolonged BI: n. a | Cardiopulmonary resuscitation: 4% | 2019 Jan 1;93(1):48-56. |
| CP: 0.51% (68/13339) | DM: 41.0% | Calcification: n. a | LCx: 23.5% | Cutting balloon: n. a | IABP: n. a | Covered stent: 27.5% | After CS implantation: 93.8% | In-hospital mortality: 8.9% |  |
| Ellis G3: 55.9% of 68 patients (0.29%) | ACS: 75.0% | Type B2C: n. a | RCA: 35.3% | Stent: 22.1% | ECMO: n. a | Coil/fat embolization: 20.6% | Surgical repair success: n.a | Acute stent thrombosis: n. a |  |
| Enrolled number: 68 patients |  |  | Others: | Rotational: 3.9% | Heparin reversed: 43.1% | Surgical repair: 2.0% |  | Periprocedural MI: n. a |  |
| **Registry: ZNA Hart centrum** | Age: n. a | CTO: 14.5% | LM: 0% | Wire: 30.9% | Pericardiocentesis: 20.0% | Prolonged Balloon inflation: 100% | Overall success: n. a | Final TIMI grade 0-1: n. a | Catheter Cardiovasc Interv |
| Enrolled period: 2007-2017 | Male: 65.5% | Bifurcation: n. a | LAD: 40.0% | POBA: 25.5% | IABP: n. a | Stent implantation: 5.5% | After prolonged BI: | Cardiopulmonary resuscitation: 12.7% | 2019;93:419–425. |
| CP: 0.29% (55/19061) | DM: n. a | Calcification: n. a | LCx: 25.5% | Cutting balloon: n. a | ECMO: n. a | Covered stent: 52.7% | After CS implantation: n. a | In-hospital mortality: 7.3% |  |
| Ellis G3: 0.18% (36/19061) | ACS: n. a | Type B2C: n. a | RCA: 21.8% | Stent: 43.6% |  | Coil embolization: n. a | CS delivery success: 82.8% | Acute stent thrombosis: n. a |  |
| Enrolled number: 55 patients |  |  | Others:9.1% | Rotational: n. a | Heparin reversed: n. a | Surgical repair: 7.3% | Surgical repair success: 75% | Periprocedural MI: n. a |  |
| **Registry: PK papyrus** | Age: n. a | CTO: n. a | LM: 2.5% | Wire: n. a | Pericardiocentesis: 8.8% | Prolonged Balloon inflation: 57.5% | Overall success: 96.1% | Final TIMI grade 0-1: n. a | Catheter Cardiovasc Interv |
| Enrolled period: unknown | Male: n. a | Bifurcation: n. a | LAD: 48.8% | POBA: n. a | IABP: n. a | Stent implantation: 8.8% | After prolonged BI: n. a | Cardiopulmonary resuscitation: n. a | 2019;94:564–568. |
| CP: n.a | DM: n. a | Calcification: .n. a | LCx: 21.3% | Cutting balloon: n. a | ECMO: n. a | Covered stent: 100% | After CS implantation: 91.3% | In-hospital mortality: 10.0% |  |
| Ellis G3: 25% of PK papyrus | ACS: n. a | Type B2C: n. a | RCA: 23.8% | Stent: n. a |  | Coil embolization: 2.5% | CS delivery success: 95.0% | Acute stent thrombosis: 1.3% |  |
| Enrolled number: 80 patients using PK papyrus |  |  | Others: 3.8% | Rotational: n. a | Heparin reversed: 1.3% | Surgical repair: 0% | Surgical repair success: n. a | Periprocedural MI: 0% |  |
| **Registry: G3-CAP (Cardio group VI)** | Age: 70.0 | CTO: 21.6% | LM: 2.0% | Wire: 18.6% | Pericardiocentesis: 27.5% | Prolonged Balloon inflation: 72.5% | Overall success: 86.3% | Final TIMI grade 0-1: 34.3% | Catheter Cardiovasc Interv. |
| Enrolled period: 1993-2015 | Male:75.5% | Bifurcation: n. a | LAD: 34.3% | POBA: 57.8% | IABP: 14.7% | Stent implantation: 0% | After prolonged BI: n. a | Cardiopulmonary resuscitation: 18.6% | 2018 Dec 1;92(7):1247-1255. |
| Ellis G3: 0.23% (224/97779) | DM:31.4% | Calcification: n. a | LCx:18.6% | Cutting balloon: 3.6% | ECMO: n. a | Covered stent: 100% | After CS implantation:86.3% | In-hospital mortality: 14.7% |  |
| Enrolled number: 102 patients | ACS: 46.1% | Type B2C: 88.2% | RCA: 26.5% | Stent: 32.5% |  | Coil embolization: 4.9% | Surgical repair success: 0% | Acute stent thrombosis: 3.9% |  |
| 102 patients using CS |  |  | Others:6.8% | Rotational: 4.8% | Heparin reversed: 35.3% | Surgical repair: 6.9% |  | Periprocedural MI:35.3% |  |
| **Registry: Hernández M et al.** | Age: 77.0 | CTO: 21.3% | LM: 3.3% | Wire: 1.6% | Pericardiocentesis: 37.7% | Prolonged Balloon inflation: n. a | Overall success: 75.4% | Cardiopulmonary resuscitation: 18.0% | J Interv Cardiol 2018;31:617–623. |
| Enrolled period: 2012-2017 | Male: 75.4% | Bifurcation: n. a | LAD: 54.1% | POBA: 19.6%, post:29.5% | IABP: 6.6% | Stent implantation: n. a | After prolonged BI: n. a | In-hospital mortality: 18.0% |  |
| CP: n. a | DM: 32.7% | Calcification: 63.9% | LCx: 14.8% | Cutting balloon: n.a | ECMO: 3.3% | Covered stent: 100% | After CS implantation: 75.4% | Acute stent thrombosis: 1.6% |  |
| Ellis G3: 90% of 61 patients | ACS: 37.7% | Type B2C: 86.9% | RCA:27.9% | Stent: 44.3% |  | Coil embolization: n. a | CS delivery success: 93.4% | Periprocedural MI: 6.6% |  |
| Enrolled number: 61 patients using CS |  |  | Others: n. a | Rotational: 4.9% | Heparin reversed: 16.4% | Surgical repair: 11.5% | Surgical repair success: n. a | SB occlusion: 9.8% |  |
| **Registry: Thorax center** | Age: 65.6 | CTO: 31.3% | LM: 1.4% | Wire: n. a | Pericardiocentesis: 48.0% | Prolonged Balloon inflation: 7.3% | Overall success: n. a | Final TIMI grade 0-1: n. a | J Am Heart Assoc. |
| Enrolled period: 2005-2016 | Male: 62.7% | Bifurcation: n. a | LAD: 39.0% | POBA: n. a | IABP: n. a | Stent implantation: 0% | After prolonged BI: n.a: | Cardiopulmonary resuscitation: n. a | 2017 Sep 22;6(9):e007049. |
| CP: 0.71% (150/21212) | DM: n. a | Calcification: 61.6% | LCx: 19.1% | Cutting balloon: 0.7% | ECMO: n. a | Covered stent: 24.0% | After CS implantation: n. a | In-hospital mortality: 8.0% |  |
| Ellis G3: 56.7% | ACS: 54.7% | Type B2C: 95.9% | RCA:36.2% | Stent: 75.3% |  | Coil/fat embolization: 2.7% | Surgical repair success: n. a | Acute stent thrombosis: n. a |  |
| Enrolled number: 150 patients |  |  | Others: 4.3% | Rotational: 2.7% | Heparin reversed: 13.7% | Surgical repair: 12.7% CABG 5.3% |  | Periprocedural MI: 34.0% |  |
| **Registry: New-Tokyo by Kawamoto** | Age: 73.6 | CTO: 42.9% | LM:7.1% | Wire: 21.4% | Pericardiocentesis: n. a | Prolonged Balloon inflation: n. a | Overall success: n. a | Final TIMI grade 0-1: n. a | Am J Cardiol. |
| Enrolled period: 2004-2013 | Male: 66.7% | Bifurcation: n. a | LAD: 45.2% | POBA: 31.0% | IABP: n. a | Stent implantation: n. a | After prolonged BI: n. a | Cardiopulmonary resuscitation: n. a | 2015 Dec 15;116(12):1822-6. |
| CS: 0.2% (42/19270), CS for Ellis G3: 35.7% | DM: 31.0% | Calcification: n. a | LCx: 14.3% | Cutting balloon: n.a | ECMO: n. a | Covered stent: 100% | After CS implantation: 83.3% | In-hospital mortality: 9.3% |  |
| Enrolled number: 42 patients | ACS: 4.8% | Type B2C: 90.4% | RCA: 33.3% | Stent: 35.7% |  | Coil embolization: n. a | CS delivery success: n. a | Acute stent thrombosis: n. a |  |
| 42 patients underwent CS implantation |  |  | Others: 0% | Rotational: 11.9% | Heparin reversed: n. a | Surgical repair: 16% | Surgical repair success: 85.7% | Periprocedural MI: n. a |  |
| **Registry: Milan-Colombo** | Age: 66.5 | CTO: 28.6% | LM: 0% | Wire: 17.9% | Pericardiocentesis: 28.6% | Prolonged Balloon inflation: 58.9% | Overall success: 87.7% | Final TIMI grade 0-1: n. a | JACC Cardiovasc Interv. |
| Enrolled period: 1993-2009 | Male: 78.6% | Bifurcation: n. a | LAD 44.6% | POBA: 50.0% | IABP: 19.6% | Stent implantation: 17.9% | After prolonged BI: 54.5% | Cardiopulmonary resuscitation: 19.6% | 2011 Jan;4(1):87-95. |
| CP: n. a | DM: 14.3% | Calcification: 23.2% | LCX 12.5%% | Cutting balloon: 7.1% | ECMO: n. a | Covered stent: 46.4% | After CS implantation: 84.6% | In-hospital mortality: 14.8% |  |
| Ellis G3: 0.23% (56/24465) | ACS: 7.1% | Type B2C: 96.4% | RCA 23.2% | Stent: 17.8% |  | Coil embolization: 1.8% | Surgical repair success: 44.4% | Acute stent thrombosis: 1.9% |  |
| Enrolled number: 56 patients |  |  | Others 19.7% | Rotational: 3.6%  DCA: 3.6% | Heparin reversed: 42.9% | Surgical repair: 16.0% |  | Periprocedural MI: 42.9% |  |

Values are numbers (%) or mean ± SD.

CP, Coronary perforation; Ellis grade III, G3; MCS, Mechanical circulatory support; DM, Diabetes mellitus; ACS, Acute coronary syndrome; CTO, chronic total occlusion; LM, left main trunk; LAD, left anterior descending artery; LCx, left circumflex artery; RCA, right coronary artery; POBA, plain old balloon angioplasty; IABP, intra-aortic balloon pumping; ECMO, extracorporeal membrane oxygenation; CS, covered stent; BI, balloon inflation; TIMI, Thrombolysis in myocardial infarction; MI, myocardial infarction
